# Barriers and facilitators to mental health treatment access and engagement for LGBTQA+ people with psychosis: a scoping review protocol

**DOI:** 10.1186/s13643-024-02566-5

**Published:** 2024-05-30

**Authors:** Cláudia C. Gonçalves, Zoe Waters, Shae E. Quirk, Peter M. Haddad, Ashleigh Lin, Lana J. Williams, Alison R. Yung

**Affiliations:** 1 Institute for Mental and Physical Health and Clinical Translation, Deakin University, Geelong, VIC 3220 Australia; 2 Telethon Kids Institute, University of Western Australia, Perth, WA 6009 Australia; 3 University Hospital Geelong, Barwon Health, Geelong, VIC 3220 Australia; 4 School of Population and Global Health, University of Western Australia, Perth, WA 6009 Australia; 5 School of Health Sciences, University of Manchester, Manchester, M13 9PL UK

**Keywords:** Psychosis, LGBT, Treatment access, Treatment engagement, Barriers, Facilitators, Clinical high risk, Ultra-high risk, At risk mental state, Psychotic experiences

## Abstract

**Background:**

The prevalence of psychosis has been shown to be disproportionately high amongst sexual and gender minority individuals. However, there is currently little consideration of the unique needs of this population in mental health treatment, with LGBTQA+ individuals facing barriers in accessing timely and non-stigmatising support for psychotic experiences. This issue deserves attention as delays to help-seeking and poor engagement with treatment predict worsened clinical and functional outcomes for people with psychosis. The present protocol describes the methodology for a scoping review which will aim to identify barriers and facilitators faced by LGBTQA+ individuals across the psychosis spectrum in help-seeking and accessing mental health support.

**Methods:**

A comprehensive search strategy will be used to search Medline, PsycINFO, Embase, Scopus, LGBTQ+ Source, and grey literature. Original studies of any design, setting, and publication date will be included if they discuss barriers and facilitators to mental health treatment access and engagement for LGBTQA+ people with experiences of psychosis. Two reviewers will independently screen titles/abstracts and full-text articles for inclusion in the review. Both reviewers will then extract the relevant data according to pre-determined criteria, and study quality will be assessed using the Joanna Briggs Institute (JBI) critical appraisal checklists. Key data from included studies will be synthesised in narrative form according to the Guidance on the Conduct of Narrative Synthesis in Systematic Reviews.

**Discussion:**

The results of this review will provide a comprehensive account of the current and historical barriers and facilitators to mental healthcare faced by LGBTQA+ people with psychotic symptoms and experiences. It is anticipated that the findings from this review will be relevant to clinical and community services and inform future research. Findings will be disseminated through publication in a peer-reviewed journal and presented at conferences.

**Scoping review registration:**

This protocol is registered in Open Science Framework Registries (10.17605/OSF.IO/AT6FC).

**Supplementary Information:**

The online version contains supplementary material available at 10.1186/s13643-024-02566-5.

## Background

The prevalence of psychotic disorders in the general population has been estimated to be around 0.27–0.75% [1, 2], with the lifetime prevalence of ever having a psychotic experience being estimated at 5.8% [3]. However, rates of psychotic symptoms and experiences are disproportionately high amongst LGBTQA+ populations, with non-heterosexual individuals estimated to be 1.99–3.75 times more likely to experience psychosis than their heterosexual peers [4–7]. Additionally, it has been estimated that transgender or gender non-conforming (henceforth trans) individuals are 2.46–49.7 times more likely than their cisgender peers (i.e. individuals whose gender identity is the same as their birth registered sex) to receive a psychotic disorder diagnosis [8, 9]. The increased rates of psychotic experiences noted amongst gender and sexual minorities may be explained by evidence indicating that LGBTQA+ people are also exposed to risk factors for psychosis at a far greater rate than members of the general population, such as childhood adversity [10–12], minority stress [13], discrimination [14], and stigma [15, 16]. Furthermore, there is added potential for diagnostic biases leading to over-diagnosing psychosis in gender diverse individuals, whose gender expression and dysphoria may be pathologized by mental health service providers [8].

Despite these concerning statistics, there is very little research examining the experiences of LGBTQA+ people with psychosis, and limited consideration of the unique needs these individuals may have in accessing and engaging with mental health services. While timely access to treatment has consistently been associated with better symptomatic and functional outcomes for people with psychosis [17, 18], there are often delays to treatment initiation which are worsened for LGBTQA+ individuals [19, 20]. These individuals face additional barriers to accessing adequate mental health support compared to cisgender/heterosexual people [19] and may need to experiment with several mental health services before finding culturally competent care [20]. This in turn may lead to longer duration of untreated psychosis. Additionally, there seems to be a lack of targeted support for this population from healthcare providers, with LGBTQA+ individuals with serious mental health concerns reporting higher rates of dissatisfaction with psychiatric services than their cisgender and heterosexual counterparts [7, 14, 21]. However, the extent of these differences varies across contexts [22], potentially due to improved education around stigma and LGBTQA+ issues within a subset of mental health services.

Nonetheless, stigma remains one of the highest cited barriers to help-seeking for mental health problems, particularly with regard to concerns around disclosure [23], which can be particularly challenging for people experiencing psychosis [24, 25]. Stigma stress in young people at risk for psychosis is associated with less positive attitudes towards help-seeking regarding both psychiatric medication and psychotherapy [26], potentially partly due to fears of judgement and being treated differently by service providers [27]. This issue may be compounded for people who also belong to minoritized groups [23, 28], particularly as LGBTQA+ individuals have reported experiencing frequent stigma and encountering uninformed staff when accessing mental healthcare [7, 29]. Furthermore, stigma-fuelled hesitance to access services may be heightened for trans people [30] whose identities have historically been pathologized and conflated with experiences of psychosis [31].

Even when individuals manage to overcome barriers to access support, there are added challenges to maintaining adequate treatment engagement. In a large online study, half of trans and nearly one third of LGB participants reported having stopped using mental health services in the past because of negative experiences related to their gender identity or sexuality [20]. This can be particularly problematic as experiences of stigma predict poorer medication adherence in psychosis [32] which subsequently multiplies the risk for relapse and suicide [33]. While no research to date has explored non-adherence rates in people with psychosis who are LGBTQA+, concerns around suicidality are heightened for individuals who are gender and sexuality diverse [34–36].

Generally, there is rising demand for mental healthcare that specifically addresses the needs of gender and sexual minority individuals and promotes respect for diversity, equity, and inclusion [29, 37]. This is particularly salient as positive relationships with staff are associated with better medication adherence for people with psychosis [38] and healthcare providers with LGBTQA+-specific mandates have demonstrated higher satisfaction rates for LGBTQA+ individuals [20]. Mental health services need to adapt treatment options to acknowledge minority stress factors for those with stigmatised identities and, perhaps more importantly, how these intersect and interact to increase inequalities in people from minoritized groups accessing and benefiting from treatment [37, 39].

Additionally, gender affirming care needs to be recognised as an important facet of mental health treatment for many trans individuals, as it is associated with positive outcomes such as improvements in quality of life and psychological functioning [40–42] and reductions in psychiatric symptom severity and need for subsequent mental health treatment [8, 43]. While there are additional barriers in access to gender affirming care for individuals with psychosis, this treatment has shown success in parallel with treatment to address psychosis symptom stabilisation [19, 44]. The importance of affirmation is echoed by the finding that many negative experiences of LGBTQA+ participants with mental health services could be avoided simply by respecting people’s pronouns and using gender-neutral language [20].

To ensure timely access to appropriate treatment for LGBTQA+ people with psychosis, there is a need for improved understanding of the factors which challenge and facilitate help-seeking and engagement with mental health support. A preliminary search of Google Scholar, Medline, the Cochrane Database of Systematic Reviews, and PROSPERO was conducted and revealed no existing or planned reviews exploring benefits and/or obstacles to mental health treatment specific to this population. Therefore, the proposed review seeks to comprehensively search and appraise the existing literature to identify and summarise a range of barriers and facilitators to adequate mental health support faced by LGBTQA+ people with experiences of psychosis. This will allow for the mapping of the types of evidence available and identification of any knowledge gaps. Moreover, we hope to guide future decision-making in mental healthcare to improve service accessibility for LGBTQA+ individuals with psychosis and to set the foundations for future research that centres this marginalised population. Based on published guidance [45–47], a scoping review methodology was identified as the most appropriate approach to address these aims.

## Methods

### Selection criteria

This scoping review protocol has been developed in compliance with the JBI Manual for Evidence Synthesis [48] and, where relevant, the Preferred Reporting Items for Systematic Review and Meta-Analysis Protocols (PRISMA-P) checklist [49] (see Additional file 1). In the event of protocol amendments, the date, justification, and description for each amendment will be provided.

Due to the limited literature around the topic of this review, any primary original study design, setting, and publication date will be considered for inclusion. Publications written in English will be included, and articles in other languages may be considered pending time and cost constraints around translation. Publications will be excluded if the full text is not available upon request from authors.

The PCC (Population, Concept, Context) framework was used to develop the inclusion criteria for this scoping review:

#### Population

This review will include individuals of any age who are LGBTQA+ and have had experiences of psychosis. For the purposes of this review, ‘LGBTQA+ individuals’ will be broadly defined as any individual that is not heterosexual and/or cisgender or anyone who engages in same-gender sexual behaviour. Studies may include participants who are cisgender and heterosexual if they separately report outcomes for LGBTQA+ individuals. Within this review, the term ‘psychosis’ includes (i) any diagnosis of a psychotic disorder, such as schizophrenia spectrum disorders, mood disorders with psychotic features, delusional disorders, and drug-induced psychotic disorders, (ii) sub-threshold psychotic symptoms, such as those present in ultra-high risk (UHR), clinical high risk (CHR), or at risk mental state (ARMS) individuals, and (iii) any psychotic-like symptoms or experiences. Studies may include participants with multiple diagnoses if they separately report outcomes for individuals on the psychosis spectrum.

#### Concept

This review will include publications which discuss potential barriers and/or facilitators to mental health help-seeking and/or engagement with mental health treatment. ‘Barriers’ will be operationalised as any factors which may delay or prevent individuals from accessing and engaging with appropriate mental health support. These may include lack of mental health education, experienced or internalised stigma, experiences of discrimination from health services, and lack of inclusivity in health services. ‘Facilitators’ will be operationalised as any factors which may promote timely help-seeking and engagement with sources of support. These may include improved access to mental health education, positive sources of social support, and welcoming and inclusive services. Mental health help-seeking will be broadly defined as any attempt to seek and access formal or informal support to address a mental health concern related to experiences of psychosis (e.g. making an initial appointment with a service provider, seeking help from a friend). Mental health treatment engagement will be broadly defined as adherence and active participation in the treatment that is offered by a source of support (e.g. attending scheduled appointments, taking medication as prescribed, openly communicating with service providers).

#### Context

This review may include research encompassing any setting in which mental healthcare is provided. This is likely to include formal healthcare settings such as community mental health teams or inpatient clinics as well as informal settings such as LGBTQA+ spaces or informal peer support. Studies will be excluded if they focus exclusively on physical health treatment.

### Search strategy

Database searches will be conducted in Medline, PsycINFO, Embase, Scopus, and LGBTQ+ Source. The full search strategy for this protocol is available (see Additional file 2). This strategy has been collaboratively developed and evaluated by a scholarly services health librarian. Searches will include subject headings relevant to each database and title/abstract keywords relating to three main concepts: (i) LGBTQA+ identity, (ii) experiences of psychosis, and (iii) mental health treatment. Keywords for each concept will be combined using the Boolean operator ‘OR’, and the three concepts will be combined using ‘AND’. This search strategy was appropriately translated for each of the selected databases. There will be no limitations on language or publication date at this stage to maximise the breadth of the literature captured. Publications returned from these searches will be exported to EndNote. Searches will be re-run prior to the final analysis to capture any newly published studies.

The database searches will be supplemented by searching the grey literature as per the eligibility criteria detailed above. These may include theses and dissertations, conference proceedings, reports from mental health services, and policy documents from LGBTQA+ groups. Google and Google Scholar will be searched using a combination of clauses for psychosis (Psychosis OR psychotic OR schizophrenia OR schizoaffective), treatment (treatment or “help-seeking”), and queer identity. The latter concept will have three clauses for three separate searches, with one including broad queer identity (LGBT), one specific to non-heterosexual individuals (gay OR lesbian OR homosexual OR bisexual OR queer OR asexual), and one specific to trans individuals (transgender OR transsexual OR transexual OR “non-binary” OR “gender minority”). Additionally, reference lists and citing literature will be manually searched for each paper included in the review to capture any articles and policy documents not previously identified.

### Data selection

Search results will be imported into Covidence using EndNote, and duplicates will be eliminated. Titles and abstracts will be screened by the first and second authors according to pre-defined screening criteria, which will be discussed by the authors and piloted prior to screening. These criteria will consider whether the articles included LGBTQA+ participants with experiences of psychosis (as operationalised above) in relation to mental health help-seeking and/or treatment. Full texts of relevant articles will then be obtained and screened by the first and second reviewer in accordance with the full inclusion and exclusion criteria after initial piloting to maximise inter-rater reliability. Decisions on inclusion and exclusion will be blinded and recorded on Covidence. Potential discrepancies will be resolved through discussion, and when consensus cannot be reached, these will be resolved by the supervising author. The process of study selection will be documented using the Preferred Reporting Items for Systematic Reviews and Meta-Analyses (PRISMA) flow diagram [50].

### Data extraction

Data extraction will be performed independently by two reviewers using Covidence. Prior to beginning final extraction, both reviewers will independently pilot the extraction tool using a sample of five included studies and discuss any necessary changes. Information extracted is planned to include the following: title, author name(s), year of publication, country in which the study was conducted, study design, sample size, population of focus (i.e. sexual minorities, gender minorities, or both), sample demographics (i.e. age, gender identity, and sexual orientation), setting (e.g. early intervention service, community mental health team, etc.), psychosis characteristics (e.g. diagnoses included, severity of symptoms, etc.), type of treatment (e.g. cognitive behavioural therapy, antipsychotic medication, etc.), and any barriers and/or facilitators identified according to the aforementioned operationalised definitions. Disagreements will be resolved through discussion between the two reviewers and, when necessary, final decisions will be made by a senior supervisor. Once extracted, information will be recorded in Excel. Lead authors of papers will be contacted by the primary review author in cases where there is missing or insufficient data.

### Quality assessment

Due to the expected heterogeneity in the types of studies that may be included in this review (e.g. qualitative studies, randomised controlled trials, case control studies, case reports), the relevant revised Joanna Briggs Institute (JBI) critical appraisal checklists [51] will be used to assess risk of bias and study quality for each study design. Two reviewers will independently use these checklists to assess each paper that is included following the full-text screening. If there are discrepancies in article ratings, these will be resolved through discussion between the two authors. If no consensus is reached, discrepancies will be resolved by a senior supervisor. In line with the scoping nature of this review, low-quality studies will not be excluded from the synthesis.

### Evidence synthesis

Data from included studies will be synthesised using a narrative synthesis approach in accordance with the *Guidance on the Conduct of Narrative Synthesis in Systematic Reviews* [52]. A preliminary descriptive synthesis will be conducted by tabulating the extracted data elements from each study alongside quality assessment results and developing an initial description of the barriers and facilitators to (1) accessing and (2) engaging with mental health support that are identified in the literature. This initial synthesis will then be interrogated and refined to contextualise these barriers and facilitators in the setting, population, and methodology of each study to form the basis for an interpretative synthesis.

This review will not use a pre-existing thematic framework to categorise barriers and facilitators as it is expected that the factors identified will not neatly fit into existing criteria. Instead, these will be conceptualised according to overarching themes as interrelated factors, so that potentially complex interactions between barriers and facilitators within and across relevant studies may be explored through concept mapping. If most of the studies included are qualitative, there may also be scope for a partial meta-synthesis. To avoid oversimplifying the concept of ‘barriers and facilitators’ (see criticism by Bach-Mortensen & Verboom [53]), this data synthesis will be followed by a critical reflection of the findings through the lens of the socio-political contexts which may give rise to the barriers and facilitators identified, exploring the complexities necessary for any changes to be implemented in mental health services.

If the extracted data indicate that gender minority and sexual minority individuals experience unique or different barriers and/or facilitators to each other, these population groups will be analysed separately as opposed to findings being generalised across the LGBTQA+ spectrum. Furthermore, if there is scope to do so, analyses may be conducted to investigate how perceived barriers and facilitators for this population may have changed over time (i.e. according to publication date) as definitions of psychosis evolve and LGBTQA+ individuals gain visibility in clinical services.

## Discussion

The proposed review will add to the literature around mental health treatment for LGBTQA+ people with psychosis. It will provide a thorough account of the barriers and facilitators to accessing and engaging with support faced by this population and may inform future research and clinical practice.

In terms of limitations, this review will be constrained by the existing literature and may therefore not be sufficiently comprehensive in reflecting the barriers and facilitators experienced by subgroups within the broader LGBTQA+ community. Additionally, although broad inclusion criteria are necessary to capture the full breadth of research conducted in this topic, included studies are likely to be heterogeneous and varied in terms of their methodology and population which may complicate data synthesis.

Nonetheless, it is anticipated that the findings from this review will provide the most comprehensive synthesis to date of the issues driving low help-seeking and treatment engagement in people across the psychosis spectrum who are LGBTQA+. This review will likely also identify gaps in the literature which may inform avenues for future research, and the factors identified in this review will be considered in subsequent research by the authors.

Additionally, findings will be relevant to healthcare providers that offer support to people with psychosis who may have intersecting LGBTQA+ identities as well as LGBTQA+ organisations which offer support to LGBTQA+ people who may be experiencing distressing psychotic experiences. These services are likely to benefit from an increased awareness of the factors which may improve or hinder accessibility for these subsets of their target populations. Therefore, results from this review may inform decision-making around the implementation of service-wide policy changes.

The findings of this review will be disseminated through the publication of an article in a peer-reviewed journal and presented at relevant conferences in Australia and/or internationally. Additionally, the completed review will form part of the lead author’s doctoral thesis.

### Supplementary Information

Additional file 1. PRISMA-P 2015 Checklist. Completed PRISMA-P Checklist for this systematic review protocol.

Additional file 2. Search Strategy. Detailed search strategy for this systematic review, including search terms and relevant controlled vocabulary terms for each included database.

## Data Availability

Not applicable for this protocol.

## Abbreviations

ARMS: At risk mental state
CHR: Clinical high risk for psychosis
JBI: Joanna Briggs Institute
LGB: Lesbian, gay, and bisexual
LGBTQA+: Lesbian, gay, bisexual, transgender, queer or questioning, asexual or aromantic, and more
PCC: Population, Concept, Context
PRISMA-P: Preferred Reporting Items for Systematic Review and Meta-Analysis Protocols
UHR: Ultra-high risk for psychosis
