# Supplementary material for: Barriers and facilitators to mental health treatment access and engagement for LGBTQA+ people with psychosis: a scoping review protocol
Source: Syst Rev. 2024 May 30;13:143. doi: 10.1186/s13643-024-02566-5 (PMC11137929; doi:10.1186/s13643-024-02566-5)
Supplement: Supplementary file 2 — Additional file 2. Search Strategy. Detailed search strategy for this systematic review, including search terms and relevant controlled vocabulary terms for each included database. [file 13643_2024_2566_MOESM2_ESM.docx]

## **SEARCH STRATEGY**

## Database name: Medline Complete (EBSCO)

| **Concept** | **Search terms** |
| --- | --- |
| LGBTQA+ identity | TI LGB* OR AB LGB*  TI queer* OR AB queer*  TI gay* OR AB gay*  TI lesbian* OR AB lesbian*  TI homosexual* OR AB homosexual*  TI bisexual* OR AB bisexual*  TI pansexual* OR AB pansexual*  TI asexual* OR AB asexual*  TI “same-sex” OR AB “same-sex”  TI “sexual minorit*” OR AB “sexual minorit*”  TI transgender* OR AB transgender*  TI transexual* OR AB transexual*  TI transsexual* OR AB transsexual*  TI “non-binary” OR AB “non-binary”  TI nonbinary OR AB nonbinary  TI “gender minorit*” OR AB “gender minorit*”  TI “gender non-conform*” OR AB “gender non-conform*”  TI “gender nonconform*” OR AB “gender nonconform*”  TI genderfluid OR AB genderfluid  TI “gender fluid” OR AB “gender fluid”  TI “two-spirit*” OR AB “two-spirit*”  TI “gender divers*” OR AB “gender divers*” |
| **OR** | |
| *Relevant controlled vocabulary term/s* | (MH "Sexual and Gender Minorities+") |
| **AND** | |
| Experiences of psychosis | TI psychos?s OR AB psychos?s  TI psychotic* OR AB psychotic*  TI schizo* OR AB schizo*  TI “clinical high risk” OR AB “clinical high risk”  TI “ultra-high risk” OR AB “ultra-high risk”  TI “at-risk mental state*” OR AB “at-risk mental state*”  TI delusion* OR AB delusion*  TI “hearing voices” OR AB “hearing voices”  TI “voice hearing” OR AB “voice hearing”  TI hallucinat* OR AB hallucinat* |
| **OR** | |
| *Relevant controlled vocabulary term/s* | (MH "Schizophrenia Spectrum and Other Psychotic Disorders+") |
| **AND** | |
| Mental health treatment | TI treatment* OR AB treatment*  TI intervention* OR AB intervention*  TI therap* OR AB therap*  TI service* OR AB service*  TI “mental healthcare” OR AB “mental healthcare”  TI “mental health care” OR AB “mental health care”  TI “help-seek*” OR AB “help-seek*”  TI “seek* help” OR AB “seek* help”  TI “seek* support” OR AB “seek* support”  TI “access* help” OR AB “access* help”  TI “access* support” OR AB “access* support”  TI medication* OR AB medication*  TI antipsychotic* OR AB antipsychotic*  TI delay* OR AB delay*  TI engage* OR AB engage*  TI complian* OR AB complian*  TI adheren* OR AB adheren*  TI barrier* OR AB barrier*  TI obstacle* OR AB obstacle*  TI challeng* OR AB challeng*  TI facilitator* OR AB facilitator*  TI enable* OR AB enable* |
| **OR** | |
| *Relevant controlled vocabulary term/s* | N/A |

## Database name: APA PsycINFO (EBSCO)

| **Concept** | **Search terms** |
| --- | --- |
| LGBTQA+ identity | TI LGB* OR AB LGB*  TI queer* OR AB queer*  TI gay* OR AB gay*  TI lesbian* OR AB lesbian*  TI homosexual* OR AB homosexual*  TI bisexual* OR AB bisexual*  TI pansexual* OR AB pansexual*  TI asexual* OR AB asexual*  TI “same-sex” OR AB “same-sex”  TI “sexual minorit*” OR AB “sexual minorit*”  TI transgender* OR AB transgender*  TI transexual* OR AB transexual*  TI transsexual* OR AB transsexual*  TI “non-binary” OR AB “non-binary”  TI nonbinary OR AB nonbinary  TI “gender minorit*” OR AB “gender minorit*”  TI “gender non-conform*” OR AB “gender non-conform*”  TI “gender nonconform*” OR AB “gender nonconform*”  TI genderfluid OR AB genderfluid  TI “gender fluid” OR AB “gender fluid”  TI “two-spirit*” OR AB “two-spirit*”  TI “gender divers*” OR AB “gender divers*” |
| **OR** | |
| *Relevant controlled vocabulary term/s* | DE "LGBTQ" OR DE "Asexuality" OR DE "Bisexuality" OR DE "Homosexuality" OR DE "Intersex" OR DE "Transgender" OR DE "Transsexualism" |
| **AND** | |
| Experiences of psychosis | TI psychos?s OR AB psychos?s  TI psychotic* OR AB psychotic*  TI schizo* OR AB schizo*  TI “clinical high risk” OR AB “clinical high risk”  TI “ultra-high risk” OR AB “ultra-high risk”  TI “at-risk mental state*” OR AB “at-risk mental state*”  TI delusion* OR AB delusion*  TI “hearing voices” OR AB “hearing voices”  TI “voice hearing” OR AB “voice hearing”  TI hallucinat* OR AB hallucinat* |
| **OR** | |
| *Relevant controlled vocabulary term/s* | DE "Psychosis" OR DE "Affective Psychosis" OR DE "Alcohol Induced Psychotic Disorders" OR DE "Brief Psychotic Disorder" OR DE "Capgras Syndrome" OR DE "Childhood Onset Psychosis" OR DE "Chronic Psychosis" OR DE "Delusional Disorder" OR DE "Experimental Psychosis" OR DE "Hallucinosis" OR DE "Paranoid Psychosis" OR DE "Postpartum Psychosis" OR DE "Reactive Psychosis" OR DE "Schizophrenia" OR DE "Substance Induced Psychotic Disorders" |
| **AND** | |
| Mental health treatment | TI treatment* OR AB treatment*  TI intervention* OR AB intervention*  TI therap* OR AB therap*  TI service* OR AB service*  TI “mental healthcare” OR AB “mental healthcare”  TI “mental health care” OR AB “mental health care”  TI “help-seek*” OR AB “help-seek*”  TI “seek* help” OR AB “seek* help”  TI “seek* support” OR AB “seek* support”  TI “access* help” OR AB “access* help”  TI “access* support” OR AB “access* support”  TI medication* OR AB medication*  TI antipsychotic* OR AB antipsychotic*  TI delay* OR AB delay*  TI engage* OR AB engage*  TI complian* OR AB complian*  TI adheren* OR AB adheren*  TI barrier* OR AB barrier*  TI obstacle* OR AB obstacle*  TI challeng* OR AB challeng*  TI facilitator* OR AB facilitator*  TI enable* OR AB enable* |
| **OR** | |
| *Relevant controlled vocabulary term/s* | N/A |

## Database name: LGBTQ+ Source (EBSCO)

| **Concept** | **Search terms** |
| --- | --- |
| LGBTQA+ identity | TI LGB* OR AB LGB*  TI queer* OR AB queer*  TI gay* OR AB gay*  TI lesbian* OR AB lesbian*  TI homosexual* OR AB homosexual*  TI bisexual* OR AB bisexual*  TI pansexual* OR AB pansexual*  TI asexual* OR AB asexual*  TI “same-sex” OR AB “same-sex”  TI “sexual minorit*” OR AB “sexual minorit*”  TI transgender* OR AB transgender*  TI transexual* OR AB transexual*  TI transsexual* OR AB transsexual*  TI “non-binary” OR AB “non-binary”  TI nonbinary OR AB nonbinary  TI “gender minorit*” OR AB “gender minorit*”  TI “gender non-conform*” OR AB “gender non-conform*”  TI “gender nonconform*” OR AB “gender nonconform*”  TI genderfluid OR AB genderfluid  TI “gender fluid” OR AB “gender fluid”  TI “two-spirit*” OR AB “two-spirit*”  TI “gender divers*” OR AB “gender divers*” |
| **OR** | |
| *Relevant controlled vocabulary term/s* | N/A |
| **AND** | |
| Experiences of psychosis | TI psychos?s OR AB psychos?s  TI psychotic* OR AB psychotic*  TI schizo* OR AB schizo*  TI “clinical high risk” OR AB “clinical high risk”  TI “ultra-high risk” OR AB “ultra-high risk”  TI “at-risk mental state*” OR AB “at-risk mental state*”  TI delusion* OR AB delusion*  TI “hearing voices” OR AB “hearing voices”  TI “voice hearing” OR AB “voice hearing”  TI hallucinat* OR AB hallucinat* |
| **OR** | |
| *Relevant controlled vocabulary term/s* | N/A |
| **AND** | |
| Mental health treatment | TI treatment* OR AB treatment*  TI intervention* OR AB intervention*  TI therap* OR AB therap*  TI service* OR AB service*  TI “mental healthcare” OR AB “mental healthcare”  TI “mental health care” OR AB “mental health care”  TI “help-seek*” OR AB “help-seek*”  TI “seek* help” OR AB “seek* help”  TI “seek* support” OR AB “seek* support”  TI “access* help” OR AB “access* help”  TI “access* support” OR AB “access* support”  TI medication* OR AB medication*  TI antipsychotic* OR AB antipsychotic*  TI delay* OR AB delay*  TI engage* OR AB engage*  TI complian* OR AB complian*  TI adheren* OR AB adheren*  TI barrier* OR AB barrier*  TI obstacle* OR AB obstacle*  TI challeng* OR AB challeng*  TI facilitator* OR AB facilitator*  TI enable* OR AB enable* |
| **OR** | |
| *Relevant controlled vocabulary term/s* | N/A |

## Database name: EMBASE

| **Concept** | **Search terms** |
| --- | --- |
| LGBTQA+ identity | LGB*:ab,ti  queer*:ab,ti  gay*:ab,ti  lesbian*:ab,ti  homosexual*:ab,ti  bisexual*:ab,ti  pansexual*:ab,ti  asexual*:ab,ti  “same-sex”:ab,ti  “sexual minorit*”:ab,ti  transgender*:ab,ti  transexual*:ab,ti  transsexual*:ab,ti  “non-binary”:ab,ti  nonbinary:ab,ti  “gender minorit*”:ab,ti  “gender non-conform*”:ab,ti  “gender nonconform*”:ab,ti  genderfluid:ab,ti  “gender fluid”:ab,ti  “two-spirit*”:ab,ti  ”gender divers*”:ab,ti |
| **OR** | |
| *Relevant controlled vocabulary term/s* | 'sexual and gender minority'/exp |
| **AND** | |
| Experiences of psychosis | psychos?s:ab,ti  psychotic*:ab,ti  schizo*:ab,ti  “clinical high risk”:ab,ti  “ultra-high risk”:ab,ti  “at-risk mental state*”:ab,ti  delusion*:ab,ti  “hearing voices”:ab,ti  ”voice hearing”:ab,ti  hallucinat*:ab,ti |
| **OR** | |
| *Relevant controlled vocabulary term/s* | 'psychosis'/exp |
| **AND** | |
| Mental health treatment | treatment*:ab,ti  intervention*:ab,ti  therap*:ab,ti  service*:ab,ti  “mental healthcare”:ab,ti  “mental health care”:ab,ti  “help-seek*”:ab,ti  “seek* help”:ab,ti  “seek* support”:ab,ti  “access* help”:ab,ti  “access* support”:ab,ti  medication*:ab,ti  antipsychotic*:ab,ti  delay*:ab,ti  engage*:ab,ti  complian*:ab,ti  adheren*:ab,ti  barrier*:ab,ti  obstacle*:ab,ti  challeng*:ab,ti  facilitator*:ab,ti  enable*:ab,ti |
| **OR** | |
| *Relevant controlled vocabulary term/s* | N/A |

## Database name: Scopus

| **Concept** | **Search terms** |
| --- | --- |
| LGBTQA+ identity | TITLE-ABS ( LGB* )  TITLE-ABS ( queer* )  TITLE-ABS ( gay* )  TITLE-ABS ( lesbian* )  TITLE-ABS ( homosexual* )  TITLE-ABS ( bisexual* )  TITLE-ABS ( pansexual* )  TITLE-ABS ( asexual* )  TITLE-ABS ( “same-sex” )  TITLE-ABS ( “sexual minorit*” )  TITLE-ABS ( transgender* )  TITLE-ABS ( transexual* )  TITLE-ABS ( transsexual* )  TITLE-ABS ( “non-binary” )  TITLE-ABS ( nonbinary )  TITLE-ABS ( “gender minorit*” )  TITLE-ABS ( “gender non-conform*” )  TITLE-ABS ( “gender nonconform*” )  TITLE-ABS ( genderfluid )  TITLE-ABS ( “gender fluid” )  TITLE-ABS ( “two-spirit*” )  TITLE-ABS ( “gender divers*” ) |
| **OR** | |
| *Relevant controlled vocabulary term/s* | N/A |
| **AND** | |
| Experiences of psychosis | TITLE-ABS ( psychos?s )  TITLE-ABS ( psychotic* )  TITLE-ABS ( schizo* )  TITLE-ABS ( “clinical high risk” )  TITLE-ABS ( “ultra-high risk” )  TITLE-ABS ( “at-risk mental state*” )  TITLE-ABS ( delusion* )  TITLE-ABS ( “hearing voices” )  TITLE-ABS ( “voice hearing” )  TITLE-ABS ( hallucinat* ) |
| **OR** | |
| *Relevant controlled vocabulary term/s* | N/A |
| **AND** | |
| Mental health treatment | TITLE-ABS ( treatment* )  TITLE-ABS ( intervention* )  TITLE-ABS ( therap* )  TITLE-ABS ( service* )  TITLE-ABS ( “mental healthcare” )  TITLE-ABS ( “mental health care” )  TITLE-ABS ( help-seek* )  TITLE-ABS ( “seek* help” )  TITLE-ABS ( “seek* support )  TITLE-ABS ( “access* help” )  TITLE-ABS ( “access* support” )  TITLE-ABS ( medication* )  TITLE-ABS ( antipsychotic* )  TITLE-ABS ( delay* )  TITLE-ABS ( engage* )  TITLE-ABS ( complian* )  TITLE-ABS ( adheren* )  TITLE-ABS ( barrier* )  TITLE-ABS ( obstacle* )  TITLE-ABS ( challeng* )  TITLE-ABS ( facilitator* )  TITLE-ABS ( enable* ) |
| **OR** | |
| *Relevant controlled vocabulary term/s* | N/A |
